# Distinguish between typical non-Hermitian quantum systems by entropy dynamics

**DOI:** 10.1038/s41598-022-06808-1

**Published:** 2022-02-18

**Authors:** Chao Zheng, Daili Li

**Affiliations:** Department of Physics, College of Science, North China University of Technology, Beijing, 100144 People’s Republic of China

**Keywords:** Quantum mechanics, Quantum simulation, Qubits, Quantum physics, Quantum information

## Abstract

Non-Hermitian (NH) quantum systems attract research interest increasingly in recent years, among which the PT-symmetric, P-pseudo-Hermitian and their anti-symmetric counterpart systems are focused much more. In this work, we extend the usage of entropy to distinguish time-evolutions of different classes and phases of typical NH-systems. In detail, we investigate the entropy dynamics of two-level NH-systems after quantum decoherence induced by single-qubit projective measurements, finding that it depends on both the initial states and the selection of the computational bases of the measurements. In a general case, we show how to distinguish all the eight phases of the above NH-systems step by step, in which process three different initial states are necessary if the basis of measurement is fixed. We propose how the distinguishing process is realized in quantum simulation, in which quantum tomography is not needed. Our investigations can be applied to judge phase transitions of non-Hermitian systems.

## Introduction

In recent years, research on non-Hermitian quantum systems^1–20^ becomes a hot area, in that it extends the conventional quantum mechanics to non-standard quantum theory^1–6^, provides links to open- and dissipative-quantum systems^21–25^, and has novel properties in application^8,26–32^. Among them are four classes of PT-symmetric systems^33–50^, P-pseudo-Hermitian systems^51–56^ and their anti-symmetric counterpart^18,19,57–66^. One important motivation studying non-Hermitian systems is that, in conventional quantum mechanics, Hermiticity is treat as a fundamental postulate to ensure that Hamiltonians have real energy eigenvalues. However, it is found to be a sufficient but not a necessary condition. One attractive extension is the PT-symmetry^1–3^. Instead of providing by the Hermitian symmetry, the eigenvalues of a PT-symmetric Hamiltonian are real if the system is in the exact PT-symmetric phase, which is separated from the PT-broken phase by the exceptional points (EPs)^67^. Further, Bender et al. defined a new inner product to develop the PT-symmetric quantum mechanics^2,3^, in which the evolution still satisfies Schrödinger’s equation. Therefore, a novel phenomenon of fast evolution with a minimal time arbitrary to zero in a PT-symmetric system is investigated both in theory^8^ and in an NMR experiment^9^. Pseudo-Hermiticity is pointed out to be a sufficient and necessary condition keeping the spectrum of Hamiltonians real^4–6^, extending the classes of NH-systems and attracting investigations^19,51–55^.

The anti-symmetric counterparts of the two classes of NH-systems start to attract interest for their appealing features, e.g., optical materials with anti-PT-photonic structures having balanced positive and negative index^57^, anti-PT-optical systems with constant refraction^62^, a diffusive system with anti-PT-symmetry^66^, etc. The exact PT (or PT-unbroken) and PT-broken phases exist in an anti-PT-symmetric system, and the phase transition occurs at the EPs, leading to many counter-intuitive phenomena. E.g., abnormal energy-difference conserving dynamics^63^, a breakdown of adiabaticity^68^, the information flow changing the direction when passing the EPs^18^, etc. Quantum simulations of anti-symmetric counterparts are also investigated^16,17,19^.

The concept of entropy is introduced by Boltzmann in the thermodynamics at first, and Shannon referred it and developed in the classical information theory. Shannon entropy is then generalized to a quantum version by von Neumann, which is still consistent with the former one for a classical uncertain state. The von Neumann entropy is well defined to the conventional Hermitian quantum system, and becomes one of the mathematical foundation of quantum information science^69^. For non-Hermitian systems, the original definition of entropy cannot be calculated directly in that the evolution is not unitary to get a non-normalized final state. Recently, Sergi et al. generalize the von Neumann entropy further^70,71^ to non-Hermitian case as a try. Wang et al. investigate the entropic uncertainty in open system^72–76^. Now that the entropy is widely used to study the disorder of a system as an important quantity, it is meaningful to discover other usages of the entropy. In this work, we extend the usage of the entropy dynamics to distinguish different phases of the four classes of non-Hermitian Hamiltonians, i.e., the PT-symmetric, P-pseudo-Hermitian, and their anti-symmetric systems of two-dimensions. We will show how to achieve the distinguishing process step by step, and propose how to realize our method in quantum simulation.

## Shannon and von Neumann entropy

While the Shannon entropy is introduced to quantify the information unpredictability of a classical system, von Neumann entropy is a general version been valid to quantum. In fact, von Neumann entropy is well defined for a pure quantum-system, a pure classical-system, and a quantum-classical hybrid-system. Therefore, it indicates the von Neumann entropy when we mention the entropy thereafter. Given that the two definitions are consistent with each other especially for classical probability distributions (e.g., states after quantum decoherence), the ellipsis of Shannon or von Neumann before entropy is unambiguous.

In a general case, no matter for a normalized or non-normalized density matrix $\rho$, von Neumann entropy can be written as

1 $$\begin{aligned} S=-tr[(\rho /tr\rho )\log _{2}(\rho /tr\rho )]. \end{aligned}$$

Consider a scenario how a classical probability distribution is obtained after quantum decoherence induced by quantum measurement. If a quantum pure state, $\rho$, is projected onto the computational bases by a measurement $\{\Pi _{k}\}$ ($k=1,2,\dots ,n$, where *n* is the dimensions of the state vector), it will collapse into one of the *n* computational bases, say $|k\rangle$. If we read out the result, the system is still in a pure state. We can realize the quantum decoherence if we do not read out the measurement result, and we will obtain a mixed state $\rho _{\mathrm{M}}$ with a classical probability distribution. For experimental realization, we can either repeat the process a plenty of times for one qubit or measure an ensemble of qubits at one time to obtain the probability distribution. It is not difficult to get the state after quantum decoherence induced by quantum measurement as

2 $$\begin{aligned} {\rho _{\mathrm{M}}}_{kj} = \delta _{kj} \rho _{kj}, \quad {\mathrm {where} \quad \delta _{kj} = \left\{ \begin{matrix} 1, &{}k=j \\ 0, &{}k\ne j \end{matrix} \right. , \quad k,j = 1,2, \ldots , n, } \end{aligned}$$

and *n* is the dimension of the quantum state. Therefore, the entropy of state ${\rho _{\mathrm{M}}}$ can be written as

3 $$\begin{aligned} S(\rho _{\mathrm{M}}) = -\sum _{k=1}^{n}\left[ tr({\rho }{\Pi }_{k}) / tr\rho \right] \log _{2}\left[ tr({\rho }{\Pi }_{k})/tr\rho \right] = -\sum _{k=1}^{n} (\rho _{kk} / \sum _{j=1}^{n} \rho _{jj}) \log _{2} (\rho _{kk} / \sum _{j=1}^{n} \rho _{jj}) \end{aligned}$$

In the single-qubit case, i.e., $n=2$, the relevant $\rho$ and $\rho _{\mathrm{M}}$ become two 2-dimensional matrices with and without non-zero off-diagonal elements, respectively. The entropy of $\rho _{\mathrm{M}}$ consists two terms

4 $$\begin{aligned} S(\rho _{\mathrm{M}})=-\left[ \rho _{11}/(\rho _{11}+\rho _{22})\right] \log _{2}\left[ \rho _{11}/(\rho _{11}+\rho _{22})\right] -\left[ \rho _{22}/(\rho _{11}+\rho _{22})\right] \log _{2}\left[ \rho _{22}/(\rho _{11}+\rho _{22})\right] . \end{aligned}$$

Noting that $\rho _{11}$ and $\rho _{22}$ may vary as time *t*, we introduce a variable

5 $$\begin{aligned} m=m(t)={\rho _{11}}/{\rho _{22}}, \end{aligned}$$

which is the ratio of the two diagonal elements of $\rho _{\mathrm{M}}$. Then $S(\rho _{\mathrm{M}}(t))$, which is a function of time *t*, can be rewritten as:

6 $$\begin{aligned} S(t)=S(\rho _{\mathrm{M}}(t))=\left( \frac{m}{1+m}\right) \log _{2}\left( 1+\frac{1}{m}\right) +\left( \frac{1}{1+m}\right) \log _{2}(1+m). \end{aligned}$$

Notice that Eq. (6) is more useful than Eq. (4), because it is well defined for both normalized and non-normalized density matrices. In the later parts, we will apply Eq. (6) to calculate the entropy of non-Hermitian systems of which the evolved density matrices are non-normalized.

## Four typical NH-systems

PT-symmetric and P-pseudo-Hermitian (PPH) systems, together with their anti-symmetric counterparts, are the most investigated NH systems for reasons that have been shown in the introduction part. We will describe the two-dimensional NH-systems in detail, which can be treat as the mathematical preparations and theoretical models to distinguish between them by their entropy dynamics after time evolutions and measurement-induced quantum decoherences.

### PT- and anti-PT-symmetric two-level systems

PT-symmetric Hamiltonians $H_{PT}$ obey the commutation with the joint operation of the parity and time-reversal operators

7 $$\begin{aligned}{}[PT, H_{PT}]=PTH_{PT}-H_{PT}PT=0, \end{aligned}$$

whereas its anti-symmetric counterpart satisfies the anti-commutation

8 $$\begin{aligned} \{PT, H_{APT}\}=PTH_{APT}+H_{APT}PT=0, \end{aligned}$$

where $H_{APT}$ is an anti-PT-symmetric (anti-PT or APT) Hamiltonian. In two-dimensional cases, the explicit forms of the two Hamiltonians can be written as

9 $$\begin{aligned} H_{PT}=\begin{pmatrix} re^{i\theta }&{}s+wi\\ s-wi&{}re^{-i\theta } \end{pmatrix} \end{aligned}$$

and

10 $$\begin{aligned} H_{APT}=i\begin{pmatrix} re^{i\theta }&{}s+wi\\ s-wi&{}re^{-i\theta } \end{pmatrix}, \end{aligned}$$

where *r*, *s*, *w* and $\theta$ are four independent real dynamic parameters. The parity operator *P* is set as $$\begin{pmatrix} 0&{}1\\ 1&{}0\end{pmatrix}$$, while *T* has the effect of complex conjugate. The eigenvalues of $H_{PT}$ and $H_{APT}$ are $\varepsilon _{\pm }=r\cos \theta \pm \sqrt{w^2+s^2-r^{2}\sin ^2\theta }$ and $i\varepsilon _{\pm }$, respectively. Thus, the energy differences of the two systems are

11 $$\begin{aligned} \Delta _{\mathrm{PT}}=2\sqrt{w^2+s^2-r^{2}\sin ^2\theta }= \Delta _{\mathrm{APT}}/i, \end{aligned}$$

which are either purely real or imaginary.

**Figure 1:**
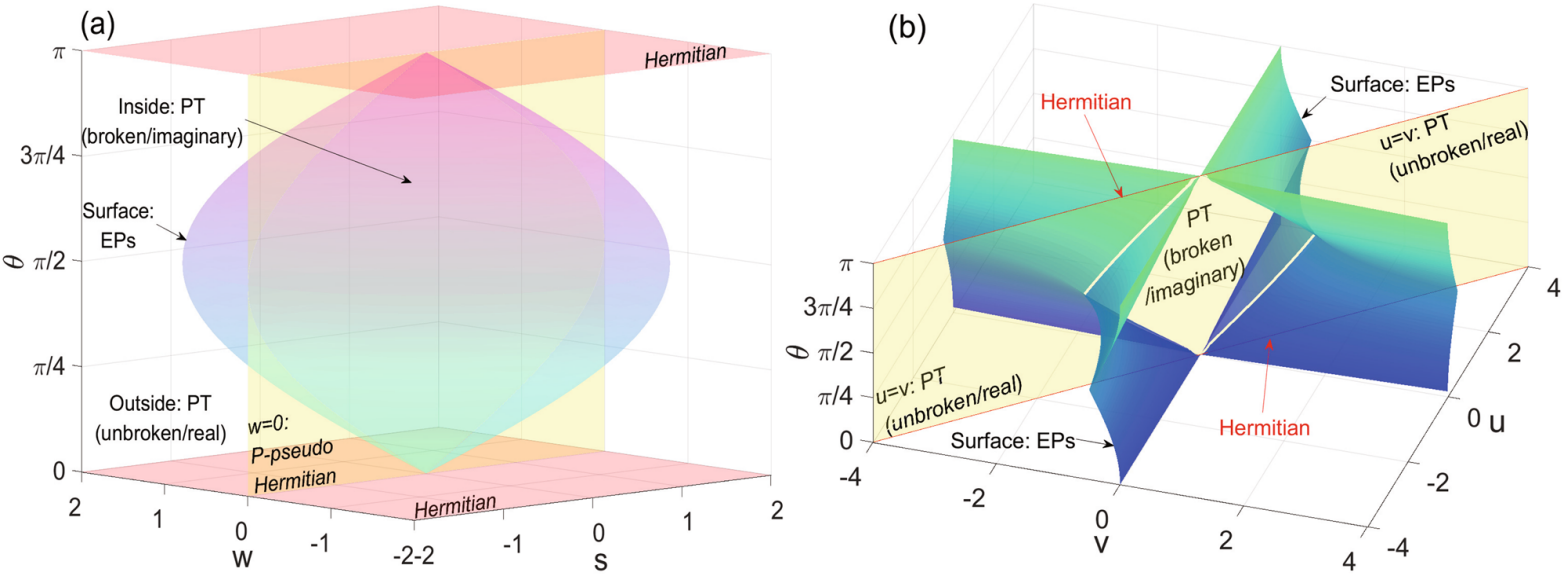
Parameter spaces of PT-symmetric and P-pseudo-Hermitian systems. From Eqs. (9) and (14), both the PT and PPH systems contain four parameters. Here we use the parameter space to illustrate the phase diagram of the two NH systems. (**a**) PT-symmetric systems. The parameter space is described by *s*, *w* and $\theta$ (here we set $r=2$). There are PT(unbroken/real) and PT(broken/imaginary) phases, EPs (the curved surface satisfying $\Delta _{PT}=0$), and intersections with PPH (the yellow plane of $w=0$) and Hermitian (pink planes of $\theta =0$ or $\pi$) systems. (**b**) P-pseudo-Hermitian systems. The parameter space is described by *u*, *v* and $\theta$ (here we set $r=2$). The saddle surface are of EPs ($\Delta _{PPH}=0$), separating $H_{PPH}$’s with real or imaginary eigenvalues which we denote as PPH(imaginary) and PPH(real) phases in the main text. The yellow plane of $u=v$ is the intersection of PPH and PT-symmetric systems.

**Figure 2:**
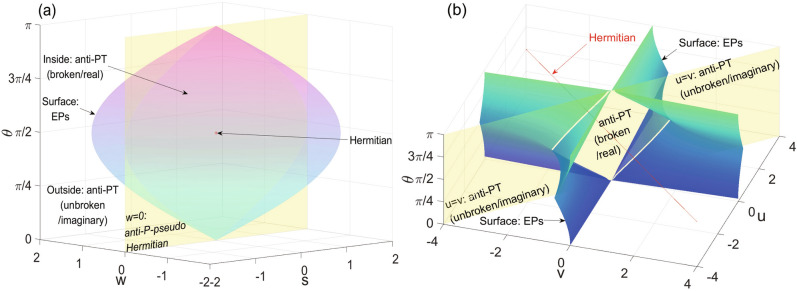
Parameter spaces of anti-PT-symmetric and anti-P-pseudo-Hermitian systems. From Eqs. (10) and (15), both the anti-PT and anti-PPH systems contain four parameters. Here we use the parameter space to illustrate the phase diagram of the two NH systems. (**a**) Anti-PT systems. The parameter space is described by *s*, *w* and $\theta$ (here we set $r=2$). There are anti-PT(unbroken/imaginary) and anti-PT(broken/real) phases, EPs (the curved surface satisfying $\Delta _{APT}=0$), and intersections with anti-PPH (the yellow plane of $w=0$) systems. (**b**) Anti-P-pseudo-Hermitian systems. The parameter space is described by *u*, *v* and $\theta$ (here we set $r=2$). The saddle surface are of EPs ($\Delta _{APPH}=0$), separating $H_{APPH}$’s with imaginary and real eigenvalue-differences which we denote as anti-PPH(imaginary) and anti-PPH(real) phases in the main text. The yellow plane of $u=v$ is the intersection of anti-PPH and anti-PT systems, while the red line indicates the intersection of anti-PPH and Hermitian systems.

For PT- or anti-PT-symmetric systems, the Hamiltonian may be exact or spontaneously broken, depending on whether the eigenstates of the Hamiltonian are also invariant under the PT-symmetry transformation or not. In the theoretical model of (anti-)PT-symmetry, we treat the two cases as PT-unbroken and PT-broken phases, which are separated by the EPs ($\Delta _\mathrm{{PT}}$ or $\Delta _\mathrm{{APT}}$ is equal to zero). Figures 1(a) and 2(a) show the two phases and EPs for the PT- and anti-PT-symmetric two-level systems, respectively. To be consistent with the (anti)P-pseudo-Hermitian systems in the next subsection, we classify the phases of this two Hamiltonians by whether the difference of eigenvalues are real or imaginary. For convenience, we denote the PT-unbroken (or broken) phase of the PT- and anti-PT-symmetric Hamiltonians as PT(real) or PT(imaginary) and anti-PT(imaginary) or antiPT(real), respectively.

### P-pseudo Hermitian two-level system and the anti-symmetric counterpart

P-pseudo Hermitian (PPH) and anti-P-pseudo Hermitian (anti-PPH or APPH) Hamiltonians, i.e. $H_{PPH}$ and $H_{APPH}$ satisfy

12 $$\begin{aligned} P\; {H_{PPH}}^{\dagger } P = H_{PPH} \end{aligned}$$

and

13 $$\begin{aligned} P\; {H_{APPH}}^{\dagger }P = -H_{APPH}, \end{aligned}$$

respectively. In two-dimensional cases, the explicit forms of them are

14 $$\begin{aligned} H_{PPH}=\begin{pmatrix} re^{i\theta }&{}v\\ u&{}re^{-i\theta } \end{pmatrix} \end{aligned}$$

and

15 $$\begin{aligned} H_{APPH}=i\begin{pmatrix} re^{i\theta }&{}v\\ u&{}re^{-i\theta } \end{pmatrix}, \end{aligned}$$

where *r*, *u*, *v* and $\theta$ are four independent real parameters; *P* is still the parity operator. The eigenvalues of $H_{PPH}$ and $H_{APPH}$ are $\varepsilon '_{\pm }=r\cos \theta \pm \sqrt{uv-r^{2}\sin ^2\theta }$ and $i\varepsilon '_{\pm }$, respectively. The energy differences of the two systems are

16 $$\begin{aligned} \Delta _{\mathrm{PPH}}=2\sqrt{uv-r^{2}\sin ^2\theta }= \Delta _{\mathrm{APPH}}/i, \end{aligned}$$

which are either purely real or imaginary. So we classify them into two phases of the (anti-)P-pseudo-Hermitian systems, denoting as PPH(real), PPH(imaginary), anti-PPH(real) and anti-PPH(imaginary), referring Figs. 1(b) and 2(b). The two phases are separated by the EPs, i.e., points lead to Eq. (16) being zero. In fact, (anti-)PT-symmetric and (anti-)P-pseudo-Hermitian systems have intersections, which is shown in Figs. 1 and 2.

## Entropy dynamics and distinguishability

**Figure 3:**
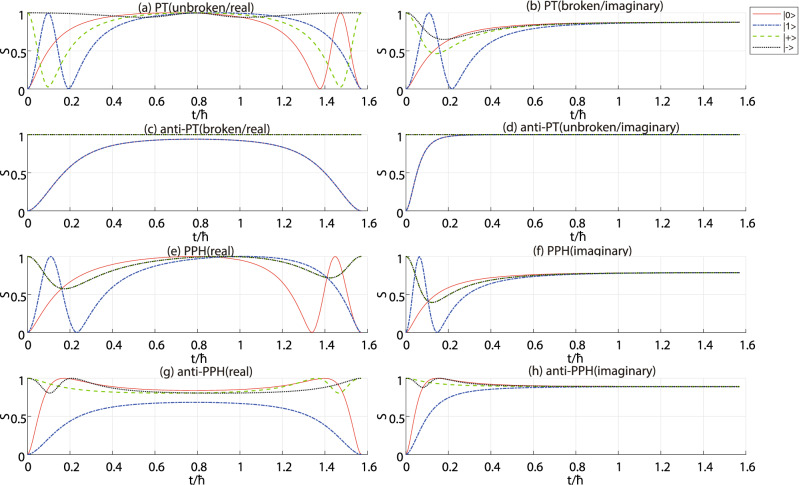
Entropy dynamics of NH systems with different input states. The computational bases are fixed as logic $|0\rangle$ and $|1\rangle$. The red solid line, blue dashed-dotted line, green dashed line and black dotted line are related to the input states $|0\rangle$, $|1\rangle$, $|+\rangle$ and $|-\rangle$, respectively. (**a**) The PT-symmetric system in PT-unbroken phase, or PT(real) phase; (**b**) The PT-symmetric system in PT-broken phase, or PT(imaginary) phase; (**c**) The anti-PT-symmetric system in PT-unbroken phase, or anti-PT(imaginary) phase; (**d**) The anti-PT-symmetric system in PT-broken phase, or anti-PT(real) phase; (**e**) The P-pseudo-Hermitian system with real eigenvalue-difference, or PPH(real) phase; (**f**) The P-pseudo-Hermitian system with imaginary eigenvalue-difference, or PPH(imaginary) phase; (**g**) The anti-P-pseudo-Hermitian system with real eigenvalue-difference, or anti-PPH(real) phase; (**h**) The anti-P-pseudo-Hermitian system with imaginary eigenvalue-difference, or anti-PPH(imaginary) phase. Notice that only one period is drawn in the subfigures (**a**), (**c**), (**e**) and (**g**), while no period in the subfigures (**b**), (**d**), (**f**) and (**h**). We set the same energy difference of the four systems in their real or imaginary phases, respectively.

The dynamic evolution of an NH system initialized in a density matrix $\rho (0)$ is governed by

17 $$\begin{aligned} \rho (t)=\frac{e^{-i\frac{t}{\hbar }H} \rho (0) e^{i\frac{t}{\hbar } H^{\dagger }}}{\text{ tr } [e^{-i\frac{t}{\hbar }H}\rho (0)e^{i\frac{t}{\hbar }H^{\dagger }}]}, \end{aligned}$$

where ${\hat{H}}$ can be one of the NH Hamiltonians in Eqs. (9), (10), (14) and (15). Given that the evolution operator $e^{-i\frac{t}{\hbar }H}$ is not unitary for the usual Hilbert-Schmidt inner product, the evolved final density matrix

18 $$\begin{aligned} \rho (t)=\begin{pmatrix} \rho _{11}&{}\rho _{12}\\ \rho _{21}&{}\rho _{22} \end{pmatrix} \end{aligned}$$

is non-normalized, where the computational basis is chosen as logic $|0\rangle$ and $|1\rangle$ without loss of generality. While $\rho (t)$ can be normalized by using $\rho _{11}+\rho _{22}$ dividing each elements, the relative probabilities or the ratio of the diagonal elements are equal. If we perform a projective measurement of a qubit in computational basis $|0\rangle$ and $|1\rangle$ on the two-level NH system, the density matrix after the measurement-induced quantum decoherence will become

19 $$\begin{aligned} \rho _{\mathrm{M}}(t)=(\rho _{11}+\rho _{22})^{-1}\begin{pmatrix} \rho _{11}&{}0\\ 0&{}\rho _{22} \end{pmatrix}, \end{aligned}$$

of which the entropy dynamics $S(\rho _{\mathrm{M}}(t))$ will be investigated as a function of time *t*. In fact, it can be calculated directly by substituting $\rho _{11}$ and $\rho _{22}$ in Eq. (18) into Eq. (6). However, the characters of the entropy dynamics vary as the input density matrix $\rho (0)$. We characterize four different cases detail in the Supplementary Information when the inputs are pure quantum states $|+\rangle$, $|-\rangle$, $|0\rangle$ and $|1\rangle$, respectively. The general patterns of entropy dynamics of the NH systems in different phases are shown in Fig. 3. Although the illustrations in the figures are with specific parameters, the patterns have general validities which can be referred to our proofs in the Supplementary Information. In the four figures in the left column Fig. 3, the patterns are periodic because their energy difference are real, and only one period is drawn there. Therefore, we denote the NH systems in their NH(real) phases when the energy difference is real, and we set the four systems have the same energy difference. Instead of the left-column subfigures in Fig. 3, patterns in the four figures in the right column of Fig. 3 have asymptotes but no period, because their energy difference are imaginary. We denote the NH systems in their NH(imaginary) phases when their energy differences are imaginary, and set the energy differences of eigenvalues the same.

Now we will show how to distinguish the eight NH systems in different phases step by step based on the entropy dynamics after time evolution and measurement-induced quantum decoherence. The quantum computational basis of the measurement is fixed to logic $|0\rangle$ and $|1\rangle$.

**Figure 4:**
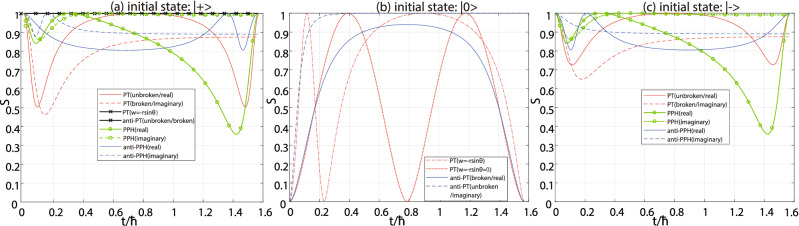
Distinguishability of the entropy dynamics of the anti-PT-symmetric and PPH systems. (**a**) With $|+\rangle$ input in the first step, the anti-PT-symmetric system can be distinguished from other systems because the entropy dynamics are constant 1 in general cases (see the black solid lines with mark ’x’). In some cases, it can also lead the entropy dynamics to be a constant 1 when the system has PT-symmetry or Hermiticity, but they can be distinguished between each other and the anti-PT-symmetric systems in different phases by inputting $|0\rangle$ further as described in the main text and illustrated in (**b**). (**b**) Distinguishablity with $|0\rangle$ input. The patterns of the four cases are typical and distinguished. (**c**) With $|-\rangle$ input in the second step, the PPH system can be identified because the entropy dynamics are identical to that with $|+\rangle$ input, which can be apparently seen when comparing the green solid or dashed line with mark ’o’ in (**a**) and that in (**c**).

In the first step, (i) $|+\rangle$ is input (referring Fig. 4(a)). If the entropy dynamics is a constant 1, the system is either the general case of anti-PT-symmetric $H_{APT}$ in PT-unbroken or -broken phase in Eq. (10) or some special cases of PT-symmetric $H_{PT}$ in Eq. (9) when $w=-r\sin \theta$. Notice that if $w=-r\sin \theta =0$ in Eq. (9), the Hamiltonian will become to a Hermitian one. (ii) Then we input $|0\rangle$ to decide that it is in anti-PT(real) phase (or PT spontaneously broken phase of anti-PT-symmetric system), anti-PT(imaginary) phase (or PT unbroken phase of anti-PT-symmetric system), some special PT-symmetric or Hermitian phases, which are illustrated in Fig. 4(b). (1) Both the patterns of entropy dynamics of the anti-PT(real) and a special Hermitian phases are periodic and are of even symmetry relevant to $t=T/2$ in each period. They can be distinguished with each other by whether the maximum reaches to 1 (the later one reaches while the former one does not). (2) The pattern of anti-PT(imaginary) phase is not periodic but increasingly approaches to 1 as the time tending to infinite. (3) the special PT-symmetric one, in which case $w=-r\sin \theta \ne 0$ in Eq. (9), is also periodic because its energy difference is always real, but has no symmetric axis at *T*/2 in each period. Therefore, these four cases in which the entropy curves are constant 1 can be distinguished between each other. If the entropy dynamics is not a constant 1 when the input state is $|+\rangle$, the distinguishing process will continue.

In the second step, $|-\rangle$ is input which is illustrated in Fig. 4(c). The entropy dynamics with $|+\rangle$ and $|-\rangle$ inputs are identical only when the system is P-pseudo Hermitian. If the entropy dynamics has a periodic pattern, the PPH system is in the PPH(real) phase. Otherwise, if the entropy dynamics is not periodic, it is in the PPH(imaginary) phase. If the entropy dynamics with $|+\rangle$ and $|-\rangle$ inputs have different patterns, the system may be either PT or anti-P-pseudo-Hermitian in different phases.

**Figure 5:**
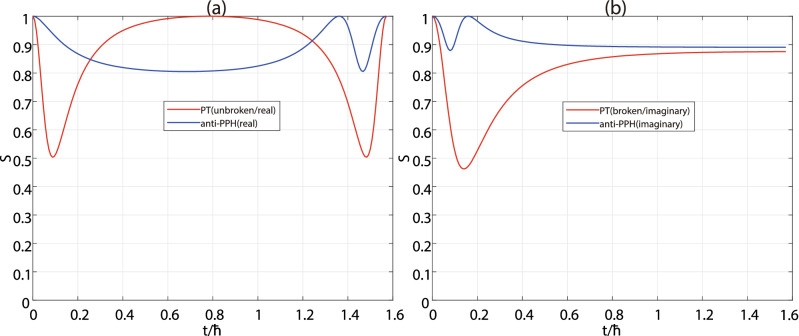
Distinguishability between the PT(real), PT(imaginary), the anti-PPH(real) and anti-PPH(imaginary) phases with $|+\rangle$. (**a**) The PT-symmetric system in unbroken or PT(real) phase is relevant to the red line, while the anti-P-pseudo Hermitian system in anti-PPH(real) phase is relevant to the blue line. Both of the entropy patterns have period *T* and only one period are drawn here. But only the former one is of even symmetry at $t=T/2$. (**b**) The PT-symmetric system in broken or PT(imaginary) phase is relevant to the red line, while the anti-P-pseudo Hermitian system in anti-PPH(imaginary) phase is relevant to the blue line. The two curves have no period but they are approaching to their respective asymptotes as the time tends to the infinite. They are distinguishable because the PT(imaginary) increasingly approaches to its asymptote, whereas the anti-PPH(imaginary) decreasingly approaches to.

In the third step, we recall the results in the first step with $|+\rangle$ input as shown in Fig. 5. (i) If the pattern of the entropy dynamics has a period *T*, and there is an axis of symmetry at $t=T/2$, the system is of PT-symmetry in the unbroken phase referring the red curve in Fig. 5(a). (ii) If the pattern of the entropy dynamics has a period *T*, and there is no axis of symmetry in each period, the system is anti-P-pseudo Hermitian with real energy difference, referring the blue curve in Fig. 5(a). If the pattern of the entropy dynamics has no period but an asymptote, the system is in PT(imaginary) or anti-PPH(imaginary) phase. (iii) The system is in PT(imaginary) phase (PT broken), if the entropy approaches to the asymptote from below when the time tends to the infinite, referring the red curve in Fig. 5(b). (iv) The system is anti-P-pseudo Hermitian with imaginary energy difference, if the pattern approach to the asymptote from above, referring the blue curve in Fig. 5(b).

## Quantum simulation and experiment proposals

**Figure 6:**
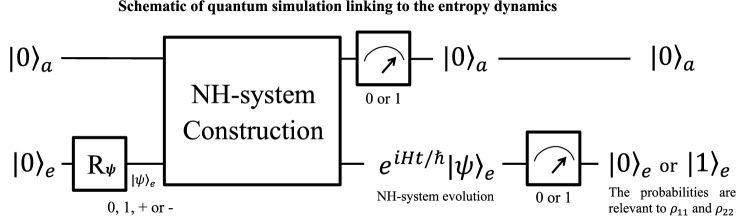
Schematic of quantum simulation to measure the entropy dynamics. The whole system is composed of an ancillary subsystem and a work qubit. For convenience, we use $|0\rangle _{a}$ to denote the initial state of the subsystems that may consist one or two qubits depending on different cases. The work qubit is initialized in $|0\rangle _{a}$ and can be rotated into one of the $|0\rangle _{e}$, $|1\rangle _{e}$ or $|\pm \rangle _{e}$ as needed to distinguish the NH systems in different phases. In the middle part, one of the NH systems will be constructed in an indeterministic way basted on the first quantum measurement. The basis of the two measurements are chosen as the logic $|0\rangle$ and $|1\rangle$. If the first output is $|0\rangle _{a}$, the result will be recorded, in which case the evolution of the work qubit is governed by one of the NH Hamiltonians. The second measurement is performed on the work qubit, and either $|0\rangle _{e}$ or $|0\rangle _{e}$ will be output. By repeating the process or inputting an ensemble of qubits, the ratio of the frequencies of outputs $|0\rangle _{a} |0\rangle _{e}$ and $|0\rangle _{a} |1\rangle _{e}$ are the experimental values of $m=\rho _{11} / \rho _{22}$, calculating the entropy by Eqs. (5) and (6).

Because the time-evolution operator $e^{-i\frac{t}{\hbar }H}$ of an NH system is nonunitary, we cannot apply the common method bing valid for a Hermitian system. One feasible method to simulate the time evolutions of NH systems is to use the linear combinations of unitaries (LCU) in the duality quantum computing scheme^77^. Quantum simulations of several NH systems have been achieved in this scheme^9,12–16,18,19^, including the four typical NH systems we investigate here. We show the schematic quantum circuit in Fig. 6, and the details can be referred in the relevant references.

We focus on how to link our investigation of entropy dynamics in this work to the previous works of quantum simulation and implementations here. We choose the logic $|0\rangle$ and $|1\rangle$ as the computational basis without loss the generality. The whole system is composed of an ancillary subsystem and a work qubit. In general, the ancillary subsystem always consists of one or two qubits, and is initialized into $|0\rangle _{a}$ or $|00\rangle _{a}$. For convenience, we use $|0\rangle _{a}$ for the two cases of the subsystem. The input state of the work qubit can be $|+\rangle _{e}$, $|-\rangle _{e}$, $|0\rangle _{e}$ or $|1\rangle _{e}$ following the steps in the former section to distinguish between the eight general cases of the four NH systems in different phases and some special PT-symmetric or Hermitian cases. Operated by the middle part of NH constructions, quantum measurements will be performed on the ancillary subsystems and the work qubit at last. If the ancillary subsystem outputs $|0\rangle _{a}$, the work qubit will evolve as $e^{-i\frac{t}{\hbar }H}$, where *H* can be one of the NH Hamiltonians in Eq. (9), (10), (12) or (13). If the outputs other than $|0\rangle _{a}$ is obtained, the result will be discarded and the simulation will be started over. The entropy at time *t* will be measured by an ensemble of the quantum simulations, in which the ratio of the frequencies of the outputs of $|0\rangle _{a}|0\rangle _{e}$ and $|0\rangle _{a}|1\rangle _{e}$ can be seen as the experimental values of $m(t)=\rho _{11} / \rho _{22}$ in Eq. (5). Substituting the two values into Eq. (6), we will get $S(\rho _M(t))$ at the moment *t*. Therefore, the entropy dynamics after quantum decoherence can be drawn by repeating this process for each moment *t*.

For experimental implementations, take the nuclear-magnetic-resonance (NMR) quantum simulator as an example, the nuclei of spin-1/2 can be treated as a qubit. The spatial-averaging method^78^ can be adopted to initialize the whole system in $|0\rangle _{a}|0\rangle _{e}$ at the beginning, and a series of magnetic pulse sequences can realize the relevant quantum gates. Specifically, a single qubit rotation can be realized by hard pulses, whereas a controlled two-qubit gate can be achieved by the free evolutions of the two nuclei of spin-$\frac{1}{2}$ in a period^9^.

Quantum optics can be another candidate, and the two orthogonal polarized directions of a photon takes a role of qubit. A single-qubit gate can be realized by a series of half-wave plates and quarter-wave plates^79^. While it is possible to realize a jointly two-qubit gate using measurement induced nonlinearity^80^, the efficiency is low in practice and improvement with the assistance of location degree of freedom should be considered^81^.

Our protocol can also be realized experimentally in other quantum devices, such as superconductor qubits, two energy levels of ultracold atoms, ion-trap systems, and etc. By noticing an interesting work^82^ to compress quantum information recently, our protocol may be realized on the IBM QE 5-qubit quantum processor using the similar method.

## Conclusions

We investigate quantum entropy dynamics of typical non-Hermitian systems in different phases, i.e., the PT- and anti-PT-symmetric two-level systems in PT-unbroken and -broken phases, the P-pseudo Hermitian and its anti-symmetric systems in real- and imaginary-eigenvalue phases. When the computational bases of the quantum measurement is fixed, three different input states are necessary to be used during the distinguishing progress in general, while less input states are enough in some special cases. Theoretically, we need to obtain the classically uncertain mixed-state by measuring the system without reading out the results. In practice, a plenty of Identical measurements will be performed to obtain the probabilities collapsing into the two computational basis as the diagonal element of the normalized density matrix of the mixed state. Because quantum tomography is not essential, our method can be realized easily in quantum simulation process by qubit-ensembles, such as an NMR system. We expect the experimental implementations in the near future. Since our method is able to distinguish eight different kinds of typical NH systems, it can be applied to judge the phase transitions of NH systems, and it maybe provide novel encoding methods for quantum communications, quantum secret sharing, and etc.

## Supplementary Information

Supplementary Information.
