# Supplementary material for: Distinguish between typical non-Hermitian quantum systems by entropy dynamics
Source: Sci Rep. 2022 Feb 18;12:2824. doi: 10.1038/s41598-022-06808-1 (PMC8857250; doi:10.1038/s41598-022-06808-1)
Supplement: Supplementary file 1 — Supplementary Information. [file 41598_2022_6808_MOESM1_ESM.pdf]

Supplementary Information: Distinguish between typical non-Hermitian quantum systems by entropy dynamics

Chao Zheng* **and Daili Li**

Department of Physics, College of Science, North China University of Technology, Beijing 100144, P. R. China.
*czheng@ncut.edu.cn

S1 Patterns of entropy dynamics of NH systems with different input states.

S1.1 $\rho(0) = |0\rangle\langle 0| = \begin{pmatrix} 1 & 0 \\ 0 & 0 \end{pmatrix}$.

The entropy dynamics of the different NH-systems are drawn analytically in Fig. 3 in the main text. Although the parameters are fixed for illustrations, we have prove that the characters of the curves of entropy dynamics are general for the relevant NH-systems. For PT-symmetric systems, (a) in PT(real) or unbroken phase, the entropy dynamics is periodical and reach the maximum 1 and the minimum 0 twice in each period, referring Fig. 3 in the main text(a); (b) in PT(imaginary) or broken phase, the entropy dynamics is approaching to its asymptote $S = S_m$ less than 1 from below, referring Fig. 3 in the main text(b). For anti-PT-symmetric systems, (c) in anti-PT(real) or broken phase, the entropy dynamics is periodical and reach the maximum less than 1 and the minimum 0 once in each period, referring Fig. 3 in the main text(c); (d) in anti-PT(imaginary) or unbroken phase, the entropy dynamics monotonically increase to its asymptote $S = 1$, referring Fig. 3 in the main text(d). For P-pseudo-Hermitian systems, (e) in PPH(real) phase, the entropy dynamics is periodical and reaches the maximum 1 and the minimum 0 twice in each period, referring Fig. 3 in the main text(e); (f) in PPH(imaginary) phase, the entropy dynamics is approaching to its asymptote, referring Fig. 3 in the main text(f). For anti-P-pseudo-Hermitian systems, (g) in anti-PPH(real) phase, the entropy dynamics is periodical, and is either like that of the anti-PT(real) phase or reach the maximum 1 twice in each period as in Fig. 3 in the main text(g); (h) in anti-PPH(imaginary) phase, the entropy dynamics either monotonically increases or firstly increases to $S = 1$ and then decreases, approaching to the asymptote, referring Fig. 3 in the main text(h).

S1.2 $\rho(0) = |1\rangle\langle 1| = \begin{pmatrix} 0 & 0 \\ 0 & 1 \end{pmatrix}$.

The entropy dynamics of the different NH-systems are drawn analytically in Fig. 3 in the main text. Although the parameters are fixed for illustrations, we have prove that the characters of the curves of entropy dynamics are general for the relevant NH-systems. For PT-symmetric systems, (a) in PT(real) or unbroken phase, the entropy dynamics is periodical and reaches the maximum 1 and the minimum 0 twice in each period, referring Fig. 3 in the main text(a); (b) in PT(imaginary) or broken phase, the entropy dynamics is approaching to its asymptote $S = S_m$ less than 1 from below, referring Fig. 3 in the main text(b). For anti-PT-symmetric systems, (c) in anti-PT(real) broken phase, the entropy dynamics has the same pattern of the entropy dynamics with S1.1, referring Fig. 3 in the main text(c); (d) in anti-PT(imaginary) or unbroken phase, the entropy dynamics has the same pattern of the entropy dynamics with S1.1, referring Fig. 3 in the main text(d). For P-pseudo-Hermitian systems, (e) in PPH(real) phase, the entropy dynamics is periodical and reach the maximum 1 and the minimum 0 twice in each period, referring FIg. 3 in the main text(e); (f) in PPH(imaginary) phase, the entropy dynamics is approaching to its asymptote, referring Fig. 3 in the main text(f). For anti-P-pseudo-Hermitian systems, (g) in anti-PPH(real) phase, the entropy dynamics is periodical, and is either like that of the anti-PT(real) phase or reach the maximum 1 twice in each period as in Fig. 3 in the main text(g); (h) in anti-PPH(imaginary) phase, entropy dynamics has the same pattern of the entropy dynamics with S1.1.

S1.3 $\rho(0) = |+\rangle\langle+| = \frac{1}{2}\begin{pmatrix} 1 & 1 \\ 1 & 1 \end{pmatrix}$.

The entropy dynamics of the different NH-systems are drawn analytically in Fig. 3 in the main text. Although the parameters are fixed for illustrations, we have prove that the characters of the curves of entropy dynamics are general for the relevant NH-systems. For PT-symmetric systems, (a) in PT(real) or unbroken phase, the entropy dynamics is periodical , reach the maximum 1 twice and is reflection symmetry in each period, referring Fig. 3 in the main text(a); (b) in PT(imaginary) or broken phase, the entropy dynamics decreases to the minimum and then increases, approaching to its asymptote $S = S_m$ less than 1, referring Fig. 3 in the main text(b). For anti-PT-symmetric systems, (c) in anti-PT(real) or broken phase, the entropy dynamics is unchanging and equals 1, referring Fig. 3 in the main text(c); (d) in anti-PT(imaginary) or unbroken phase, the entropy dynamics is unchanging and equals 1, referring Fig. 3 in the main text(d). For P-pseudo-Hermitian systems, (e) in PPH(real) phase, the entropy dynamics is periodical and reaches 1 twice in each period, referring FIg. 3 in the main text(e); (f) in PPH(imaginary) phase, the entropy dynamics is approaching to its asymptote , referring FIg. 3 in the main text(f). For anti-P-pseudo-Hermitian systems, (g) in anti-PPH(real) phase, the entropy dynamics is periodical and reaches 1 twice, referring Fig. 3 in the main text(g); (h) in anti-PPH(imaginary) phase, the entropy dynamics either monotonically decreases or oscillates once and then decreases, approaching to the asymptote S_m less than 1, referring Fig. 3 in the main text(h).

S1.4 $\rho(0) = |-\rangle\langle-| = \frac{1}{2}\begin{pmatrix} 1 & -1 \\ -1 & 1 \end{pmatrix}$.

The entropy dynamics of the different NH-systems are drawn analytically in Fig. 3 in the main text. Although the parameters are fixed for illustrations, we have prove that the characters of the curves of entropy dynamics are general for the relevant NH-systems. For PT-symmetric systems, (a) in PT(real) or unbroken phase, the entropy dynamics is periodical , reach the maximum 1 twice and is reflection symmetry in each period, referring Fig. 3 in the main text(a); (b) in PT(imaginary) or broken phase, the entropy dynamics has similar pattern of entropy dynamics in S1.3 (b), referring Fig. 3 in the main text(b). For anti-PT-symmetric systems, (c) in anti-PT(real) or broken phase, the entropy dynamics is unchanging and equals 1, referring Fig. 3 in the main text(c); (d) in anti-PT(imaginary) or unbroken phase, the entropy dynamics is unchanging and equals 1, referring Fig. 3 in the main text(d). For P-pseudo-Hermitian systems, (e) in PPH(real) phase, the entropy dynamics has similar pattern of entropy dynamics in S1.3 (e), referring FIg. 3 in the main text(e); (f) in PPH(imaginary) phase, the entropy dynamics has similar pattern of entropy dynamics in S1.3 (b), referring FIg. 3 in the main text(f). For anti-P-pseudo-Hermitian systems, (g) in anti-PPH(real) phase, the entropy dynamics is periodical and reaches 1 twice, referring Fig. 3 in the main text(g); (h) in anti-PPH(imaginary) phase, the entropy dynamics has similar pattern of entropy dynamics in S1.3 (h), referring Fig. 3 in the main text(h).

S2 Proof of typical characters of patterns of entropy dynamics of different NH systems with different input states.

S2.1 PT symmetry system: The time-evolution operator $e^{-i\frac{t}{\hbar}H_{PT}}$ is:

$$e^{-\frac{t}{\hbar}ir\cos\theta}\begin{pmatrix} a_0+a_3 & a_1-ia_2 \\ -a_1-ia_2 & a_0-a_3 \end{pmatrix} \tag{S1}$$

where $\Delta_{\mathrm{PT}} = \sqrt{w^2+s^2-r^2\sin^2\theta}$, $\alpha = \frac{\Delta_{\mathrm{PT}}}{2\hbar}t, a_0 = \cos\alpha$, $a_1 = 2w\sin\alpha/\Delta_{PT}$, $a_2 = 2s\sin\alpha/\Delta_{PT}$ and $a_3 = 2r\sin\theta\sin\alpha/\Delta_{PT}$, and a_j's $(j = 0,1,2,3)$are real numbers.

When the initial states are $|0\rangle$, $|1\rangle$, $|+\rangle$ and $|-\rangle$, $m(t)$ (referring Eq. (5) in the manuscript) are shown in Table S1 (the energy difference $\Delta_{\mathrm{PT}} = \Delta$ is either real or imaginary):

Table S1

$\rho(0)$	The energy difference (Δ)	$m(t)$
$\|0\rangle\langle 0\|$	real	$\frac{1}{4(s^2+w^2)}(\Delta\cot\alpha+2r\sin\theta)^2$
$\|0\rangle\langle 0\|$	imaginary	$\frac{1}{4(s^2+w^2)}(\|\Delta\|\coth\|\alpha\|+2r\sin\theta)^2$
$\|1\rangle\langle 1\|$	real	$\frac{4(s^2+w^2)}{(\Delta\cot\alpha-2r\sin\theta)^2}$
$\|1\rangle\langle 1\|$	imaginary	$\frac{4(s^2+w^2)}{(\|\Delta\|\coth\|\alpha\|+2r\sin\theta)^2}$
$\|+\rangle\langle +\|$	real	$1+\frac{8(r\sin\theta+w)\cot\alpha}{(-\Delta\cot\alpha+w+r\sin\theta)^2+4s^2}$
$\|+\rangle\langle +\|$	imaginary	$\frac{(\|\Delta\|\coth\|\alpha\|+2w+2r\sin\theta)^2+4s^2}{(-\|\Delta\|\coth\|\alpha\|+2w+2r\sin\theta)^2+4s^2}$
$\|-\rangle\langle -\|$	real	$1+\frac{8(r\sin\theta-w)\cot\alpha}{(-\Delta\cot\alpha-w+r\sin\theta)^2+4s^2}$
$\|-\rangle\langle -\|$	imaginary	$\frac{(\|\Delta\|\coth\|\alpha\|-2w+2r\sin\theta)^2+4s^2}{(-\|\Delta\|\coth\|\alpha\|-2w+2r\sin\theta)^2+4s^2}$

Claim 1. In PT(real) or unbroken phase, when $\rho(0)=|0\rangle\langle 0|$, $|1\rangle\langle 1|$, $|+\rangle\langle +|$ or $|-\rangle\langle -|$, the entropy $S(t)$ has period $T=\frac{2\pi\hbar}{\Delta}$.
Proof. Substituting $T=\frac{2\pi\hbar}{\Delta}$ into $m(t)$ in Table S1 and $S(m(t))$ in Eq.6 in the manuscript, it is easy to check that

$$m(\frac{2\pi\hbar}{\Delta}+t)=m(t) \quad \text{and} \quad S(\frac{2\pi\hbar}{\Delta}+t)=S(t). \tag{S2}$$

which means that the entropy has a period $\frac{2\pi\hbar}{\Delta}$.

Claim 2. In PT(real) phase (or PT spontaneously unbroken phase), when $\rho(0)=|0\rangle\langle 0|$, the entropy $S(t)$ will reach 0 and 1 twice in each period.
Proof. Solving t such that $S(t)=0$, we have

$$t=kT \text{ or } t=kT+\tfrac{2\hbar}{\Delta}\operatorname{arccot}\left(-\tfrac{2r\sin\theta}{\Delta}\right), \quad \text{where } k=0,1,\dots \tag{S3}$$

Solving t such that $S(t)=1$, we have

$$t=kT+\frac{2\hbar}{\Delta}\operatorname{arccot}\left(\frac{-2r\sin\theta\pm 2\sqrt{w^2+s^2}}{\Delta}\right), \quad \text{where } k=0,1,\dots \tag{S4}$$

Claim 3. In PT(real) or unbroken phase, when $\rho(0)=|+\rangle\langle +|$, the entropy $S(t)$ has an asymmetry axis at $t=T/2$(where T is the period of $S(t)$), i.e. $S(T-t)=S(t)$.
Proof. We have $m(T-t)=\frac{1}{m(t)}$, then, by substituting it into Eq. (6) in the manuscript, we have $S(T-t)=S(t)$.

Claim 4. In PT(real) or unbroken phase, when $\rho(0)=|+\rangle\langle +|$, the entropy $S(t)$ will reach 1 twice in each period.
Proof. Solving t such that $S(t)=1$, we have

$$t=kT+\frac{\hbar\pi}{\Delta} \quad \text{or} \quad t=kT, \quad \text{where } k=0,1,\dots \tag{S5}$$

Claim 5. In PT(real) or unbroken phase, when $\rho(0)=|+\rangle\langle +|$, the entropy $S(t)$ is always 1 if and only if $w=-r\sin\theta$.
Proof. Solving m such that $S(m)\equiv 1$ in Eq.6:

$$m(t)\equiv 1 \iff w=-r\sin\theta. \tag{S6}$$

Claim 6. In PT(imaginary) or broken phase, when $\rho(0)=|0\rangle\langle 0|$, $|1\rangle\langle 1|$, $|+\rangle\langle +|$ or $|-\rangle\langle -|$, the entropy $S(t)$ will approach their asymptotes, respectively.

Proof. When $\rho(0) = |0\rangle\langle 0|$, $|1\rangle\langle 1|$, $|+\rangle\langle +|$ or $|-\rangle\langle -|$, $\lim_{t\to\infty} m(t)$ are:

$$\begin{aligned} m_0 &= \frac{|\Delta|^2 + 4r|\Delta|\sin\theta + 4r^2\sin^2\theta}{4s^2+4w^2} \neq 1, \\ m_1 &= \frac{4s^2+4w^2}{|\Delta|^2 - 4r|\Delta|\sin\theta + 4r^2\sin^2\theta} \neq 1, \\ m_+ &= \frac{(|\Delta|+2w+2r\sin\theta)^2+4s^2}{(-|\Delta|+2w+2r\sin\theta)^2+4s^2} \neq 1, \\ m_- &= \frac{(|\Delta|-2w+2r\sin\theta)^2+4s^2}{(-|\Delta|-2w+2r\sin\theta)^2+4s^2} \neq 1. \end{aligned} \tag{S7}$$

Substituting $m(t\to\infty)$'s into Eq. (6) in the manuscript, we can get the relevant $S(t\to\infty)$'s are asymptotes and less than 1.

Claim 7. In PT(imaginary) or broken phase, when $\rho(0) = |+\rangle\langle +|$, the entropy $S(t)$ will approach the asymptote $S = S(m_+)$ from below.

Proof. There exists a moment t_1 such that $S(t)$ monotonically increases to $S(m_+)$ after $t > t_1$ because $\dfrac{dS(t)}{dt} > 0$ and $S(t) - S(m_+) < 0$, where

$$t_1 = \frac{\hbar}{|\Delta|}\ln\left(\frac{|\Delta| + \sqrt{4s^2+4(w+r\sin\theta)^2}}{-|\Delta| + \sqrt{4s^2+4(w+r\sin\theta)^2}}\right) \tag{S8}$$

S2.2 Anti-PT symmetry system The time-evolution operator $e^{-i\frac{t}{\hbar}H_{APT}}$ is:

$$e^{\frac{t}{\hbar}r\cos\theta}\begin{pmatrix} a_0+ia_3 & ia_1+a_2 \\ -ia_1+a_2 & a_0-ia_3 \end{pmatrix} \tag{S9}$$

where $\Delta_{\text{APT}} = \sqrt{r^2\sin^2\theta - w^2 - s^2}$, $\alpha = \frac{\Delta_{\text{APT}}}{2\hbar}t, a_0 = \cos\alpha$, $a_1 = 2w\sin\alpha/\Delta_{APT}$, $a_2 = 2s\sin\alpha/\Delta_{APT}$ and $a_3 = 2r\sin\theta\sin\alpha/\Delta_{APT}$, and a_j's $(j = 0,1,2,3)$are real numbers.

When the initial states are $|0\rangle$, $|1\rangle$, $|+\rangle$ and $|-\rangle$, $m(t)$ are shown in Table S2 (the energy difference $\Delta_{\text{APT}} = \Delta$ is either real or imaginary):

Table S2

$\rho(0)$	The energy difference (Δ)	$m(t)$
$\|0\rangle\langle 0\|$	real	$\frac{1}{4(s^2+w^2)}(\Delta^2\cot^2\alpha + 4r^2\sin^2\theta)$
$\|0\rangle\langle 0\|$	imaginary	$\frac{1}{4(s^2+w^2)}\|\Delta\|^2\coth^2\|\alpha\| + 4r^2\sin^2\theta$
$\|1\rangle\langle 1\|$	real	$\frac{4(s^2+w^2)}{\Delta^2\cot^2\alpha+4r^2\sin^2\theta}$
$\|1\rangle\langle 1\|$	imaginary	$\frac{4(s^2+w^2)}{\|\Delta\|^2\coth^2\|\alpha\|+4r^2\sin^2\theta}$
$\|+\rangle\langle +\|$	real	1
$\|+\rangle\langle +\|$	imaginary	1
$\|-\rangle\langle -\|$	real	1
$\|-\rangle\langle -\|$	imaginary	1

Claim 1. For anti-PT symmetric systems, when $\rho(0) = |+\rangle\langle+|$ and $|-\rangle\langle-|$, the entropy $S(t) \equiv 1$.
Proof. From the Table S2, it is easy to check $m(t) \equiv 1$, then we derive $S(t) \equiv 1$ by substituting $m(t)$ in to Eq. 6.

Claim 2. For anti-PT symmetric systems, the entropy $S(t)$ of $|0\rangle\langle 0|$ and $|1\rangle\langle 1|$ are same.
Proof. We set that $m_0(t)$ and $m_1(t)$ respectively represent the $m(t)$ of $|0\rangle\langle 0|$ and $|1\rangle\langle 1|$. From Table S2 we get

$$m_0(t) = \frac{1}{m_1(t)} \tag{S10}$$

$S(t)$ are same by substituting m's into Eq. (6).

Claim 3. In anti-PT(real) phase (or PT broken phase of APT system), when $\rho(0) = |0\rangle\langle 0|$, $|1\rangle\langle 1|$, $|+\rangle\langle+|$ or $|-\rangle\langle-|$, the entropy $S(t)$ has period $T = \frac{2\pi\hbar}{\Delta}$.
Proof. Substituting $T = \frac{2\pi\hbar}{\Delta}$ into $m(t)$ in Table S2 and $S(m(t))$ in Eq.6 in the manuscript, it is easy to check that

$$m(\frac{2\pi\hbar}{\Delta}+t) = m(t) \quad \text{and} \quad S(\frac{2\pi\hbar}{\Delta}+t) = S(t). \tag{S11}$$

which means that the entropy has a period $\frac{2\pi\hbar}{\Delta}$.

Claim 4. In anti-PT(real) phase (or PT broken phase of APT system), the entropy $S(t)$ of $|0\rangle\langle 0|$ or $|1\rangle\langle 1|$ have one maximum lower than 1.
Proof. The period T is $\frac{2\pi\hbar}{\Delta}$, when $t \in [0, T/2]$, $S(t)' > 0$ and when $t \in [T/2, T]$, $S(t)' < 0$. The maximum is

$$Smax = S(T/2) = S\left(\frac{r^2\sin^2\theta}{s^2+w^2}\right) < 1 \tag{S12}$$

We have same conclusion when $\rho(0) = |1\rangle\langle 1|$ because of Claim 2.

Claim 5. In anti-PT(imaginary) phase (or PT unbroken phase of APT system), the entropy $S(t)$ of $|0\rangle\langle 0|$ or $|1\rangle\langle 1|$ approach the asymptote $\lim_{t\to\infty} S(t) = 1$ from below.
Proof. We have $\lim_{t\to\infty} m(t)$ of $|0\rangle\langle 0|$ or $|1\rangle\langle 1|$ are $m_0 = m_1 = 1$ from Table S2, so we can derive $\lim_{t\to\infty} S(t) = 1$. We also have $\dfrac{dS(t)}{dt} > 0$, so $S(t)$ monotonically increases to 1.

S2.3 P-pseudo-Hermitian system

The time-evolution operator $e^{-i\frac{t}{\hbar}H_{PPH}}$ is:

$$e^{-\frac{t}{\hbar}ir\cos\theta}\begin{pmatrix} a_0+a_3 & -ia_1 \\ -ia_2 & a_0-a_3 \end{pmatrix} \tag{S13}$$

where $\Delta_{\mathrm{PPH}} = 2\sqrt{vu - r^2\sin^2\theta}$, $\alpha = \frac{\Delta_{\mathrm{PPH}}t}{2\hbar}, a_0 = \cos\alpha, a_1 = 2v\sin\alpha/\Delta_{\mathrm{PPH}}, a_2 = 2u\sin\alpha/\Delta_{\mathrm{PPH}}$ and $a_3 = 2r\sin\theta\sin\alpha/\Delta_{\mathrm{PPH}}$, $a'_js(j = 0,1,2,3)$ are real numbers.

When the initial states are $|0\rangle$, $|1\rangle$, $|+\rangle$ and $|-\rangle$, $m(t)$ are shown in Table S3 (the energy difference $\Delta_{\mathrm{PPH}} = \Delta$ is either real or imaginary):

Table S3

$\rho(0)$	The energy difference (Δ)	$m(t)$
$\|0\rangle\langle 0\|$	real	$\frac{1}{4u^2}(\Delta\cot\alpha+2r\sin\theta)^2$
$\|0\rangle\langle 0\|$	imaginary	$\frac{1}{4u^2}(\|\Delta\|\coth\|\alpha\|+2r\sin\theta)^2$
$\|1\rangle\langle 1\|$	real	$\frac{4v^2}{(\Delta\cot\alpha-2r\sin\theta)^2}$
$\|1\rangle\langle 1\|$	imaginary	$\frac{4v^2}{(\|\Delta\|\coth\|\alpha\|-2r\sin\theta)^2}$
$\|+\rangle\langle +\|$	real	$\frac{(\Delta\cot\alpha+2r\sin\theta)^2+4v^2}{(\Delta\cot\alpha-2r\sin\theta)^2+4u^2}$
$\|+\rangle\langle +\|$	imaginary	$\frac{(\|\Delta\|\coth\|\alpha\|+2r\sin\theta)^2+4v^2}{(\|\Delta\|\coth\|\alpha\|-2r\sin\theta)^2+4u^2}$
$\|-\rangle\langle -\|$	real	$\frac{(\Delta\cot\alpha+2r\sin\theta)^2+4v^2}{(\Delta\cot\alpha-2r\sin\theta)^2+4u^2}$
$\|-\rangle\langle -\|$	imaginary	$\frac{(\|\Delta\|\coth\|\alpha\|+2r\sin\theta)^2+4v^2}{(\|\Delta\|\coth\|\alpha\|-2r\sin\theta)^2+4u^2}$

Claim 1. In P-pseudo Hermitian all phases, the entropy $S(t)$ are familiar of $|+\rangle\langle +|$ and $|-\rangle\langle -|$.
Proof.

(1) From Table S3, in PPH(real) phase, $\rho(0) = |+\rangle\langle +|$ and $|-\rangle\langle -|$, it is easy to check that m's are similar , so that their entropy $S(t)$ are same too by Eq. (6).

(2) In PPH(imaginary) phase, entropy $S(t)$ of $|+\rangle\langle +|$ and $|-\rangle\langle -|$ are same for the same reason.

Claim 2. In PPH(real) phase, when $\rho(0) = |0\rangle\langle 0|$, $|1\rangle\langle 1|$, $|+\rangle\langle +|$ or $|-\rangle\langle -|$, the entropy $S(t)$ has a period $T = \frac{2\pi\hbar}{\Delta}$.
Proof. Substitute $T = \frac{2\pi\hbar}{\Delta}$ into $m(t)$ in Table S4 and $S(m(t))$ in Eq. 6 in the manuscript, it is easy to check that

$$m(\frac{2\pi\hbar}{\Delta}+t) = m(t) \quad \text{and} \quad S(\frac{2\pi\hbar}{\Delta}+t) = S(t), \tag{S14}$$

which means that the entropy has a period $\frac{2\pi\hbar}{\Delta}$.

Claim 3. In PPH(real) phase, $\rho(0) = |0\rangle\langle 0|$ and $|1\rangle\langle 1|$, the entropy $S(t)$ will reach 0 and 1 twice.
Proof.
(1) When $\rho(0) = |0\rangle\langle 0|$, the solution of t when $S(t) = 0$ are

$$t = kT + \frac{2\hbar}{\Delta}\text{arccot}\,\frac{-2r\sin\theta}{\Delta} \text{ or } t = kT, \tag{S15}$$

the solution of t when $S(t) = 1$ are

$$t = kT + \frac{2\hbar}{\Delta}\text{arccot}\,\frac{-2r\sin\theta \pm 2u}{\Delta}, \tag{S16}$$

where $k = 0, 1, \ldots$.
(2) When $\rho(0) = |1\rangle\langle 1|$, the solution of t when $S(t) = 0$ are

$$t = kT + \frac{2\hbar}{\Delta}\text{arccot}\,\frac{2r\sin\theta}{\Delta} \text{ or } t = kT, \tag{S17}$$

the solution of t when $S(t) = 0$ are

$$t = kT + \frac{2\hbar}{\Delta}\text{arccot}\,\frac{-2r\sin\theta \pm 2v}{\Delta}, \tag{S18}$$

where $k = 0, 1, \ldots$.

Claim 4. In PPH(real) phase, when $\rho(0) = |+\rangle\langle+|$, the entropy $S(t)$ will reach 1 twice in each period.
Proof. Solving t when $S(t) = 1$, we have

$$t = \frac{2\hbar}{\Delta}\operatorname{arccot}\frac{u^2-v^2}{2\Delta r\sin\theta} + kT \text{ or } t = kT., \text{ where } k = 0,1,\ldots \tag{S19}$$

Claim 5. In PPH(imaginary) phase, when $\rho(0) = |0\rangle\langle 0|$, $|1\rangle\langle 1|$, $|+\rangle\langle+|$ or $|-\rangle\langle-|$, the entropy $S(t)$ will approach their asymptotes, respectively.
Proof. When $\rho(0) = |0\rangle\langle 0|$, $|1\rangle\langle 1|$, $|+\rangle\langle+|$ or $|-\rangle\langle-|$, $\lim_{t\to\infty} m(t)$ are

$$m_0 = \frac{1}{4u^2}(|\Delta|+2r\sin\theta)^2, m_1 = \frac{4v^2}{(|\Delta|-2r\sin\theta)^2}, m_+ = m_- = \frac{(|\Delta|+2r\sin\theta)^2+4v^2}{(|\Delta|-2r\sin\theta)^2+4u^2}. \tag{S20}$$

Substituting the m's above into Eq. (6) in the manuscript, we can calculate the limit of entropy $\lim_{t\to\infty} S(t)$.
In two special cases,

$$\begin{aligned} S(m_0) = 0 &\iff (m = 0 \text{ or } m = \infty) \iff uv = 0, \\ S(m_1) = 0 &\iff (m = 0 \text{ or } m = \infty) \iff uv = 0. \end{aligned} \tag{S21}$$

S2.4 Anti-P-pseudo Hermitian system. The time-evolution operator $e^{-i\frac{t}{\hbar}H_{APPH}}$ is:

$$e^{\frac{t}{\hbar}r\cos\theta}\begin{pmatrix} a_0+ia_3 & a_1 \\ a_2 & a_0-ia_3 \end{pmatrix} \tag{S22}$$

where $\Delta_{\mathrm{APPH}} = 2\sqrt{r^2\sin^2\theta - uv}$ is the energy difference , $\alpha = \frac{\Delta_{\mathrm{APPH}}t}{2\hbar}$, $a_0 = \cos\alpha$, $a_1 = 2v\sin\alpha/\Delta_{\mathrm{APPH}}$, $a_2 = 2u\sin\alpha/\Delta_{\mathrm{APPH}}$ and $a_3 = 2r\sin\theta\sin\alpha/\Delta_{\mathrm{APPH}}$, and a_j's $(j = 0,1,2,3)$are real numbers.
When the initial states are $|0\rangle$, $|1\rangle$, $|+\rangle$ and $|-\rangle$, $m(t)$ are shown in Table S4 (the energy difference $\Delta_{\mathrm{APPH}} = \Delta$ is either real or imaginary):

Table S4

$\rho(0)$	The energy difference Δ	$m(t)$
$\|0\rangle\langle 0\|$	real	$\frac{1}{4u^2}(\Delta^2\cot^2\alpha+4r^2\sin\theta)$
$\|0\rangle\langle 0\|$	imaginary	$\frac{1}{4u^2}(\|\Delta\|^2\coth^2\|\alpha\|+4r^2\sin\theta)$
$\|1\rangle\langle 1\|$	real	$\frac{4v^2}{\Delta^2\cot^2\alpha+4r^2\sin\theta}$
$\|1\rangle\langle 1\|$	imaginary	$\frac{4v^2}{\|\Delta\|^2\coth^2\|\alpha\|+4r^2\sin\theta}$
$\|+\rangle\langle+\|$	real	$\frac{(\Delta\cot\alpha+2v)^2+4r^2\sin^2\theta}{(\Delta\cot\alpha+2u)^2+4r^2\sin^2\theta}$
$\|+\rangle\langle+\|$	imaginary	$\frac{(\|\Delta\|\coth\|\alpha\|+2v)^2+4r^2\sin^2\theta}{(\|\Delta\|\coth\|\alpha\|+2u)^2+4r^2\sin^2\theta}$
$\|-\rangle\langle-\|$	real	$\frac{(\Delta\cot\alpha-2v)^2+4r^2\sin^2\theta}{(\Delta\cot\alpha-2u)^2+4r^2\sin^2\theta}$
$\|-\rangle\langle-\|$	imaginary	$\frac{(\|\Delta\|\coth\|\alpha\|-2v)^2+4r^2\sin^2\theta}{(\|\Delta\|\coth\|\alpha\|-2u)^2+4r^2\sin^2\theta}$

Claim 1. In anti-PPH(real) phase, when $\rho(0) = |0\rangle\langle 0|$, $|1\rangle\langle 1|$, $|+\rangle\langle+|$ or $|-\rangle\langle-|$, the entropy $S(t)$ has a period $T = \frac{2\pi\hbar}{\Delta}$.
Proof. Substitute $T = \frac{2\pi\hbar}{\Delta}$ into $m(t)$ in Table S4 and $S(m(t))$ in Eq. 6 in the manuscript, it is easy to check that

$$m(\frac{2\pi\hbar}{\Delta}+t) = m(t) \quad \text{and} \quad S(\frac{2\pi\hbar}{\Delta}+t) = S(t), \tag{S23}$$

which means that the entropy has a period $\frac{2\pi\hbar}{\Delta}$.

Claim 2. In anti-PPH(real), when $\rho(0) = |0\rangle\langle 0|$ or $|1\rangle\langle 1|$, the entropy will either reach 1 twice or reach a maximum less than 1 once in each period T.
Proof. (1) $|u| < |v|$. (a) When $\rho(0) = |1\rangle\langle 1|$, there exist a moment

$$t_1 = kT + \frac{2\hbar}{\Delta}\text{arccot}\left(\pm\frac{2\sqrt{v^2 - r^2\sin^2\theta}}{\Delta}\right) \tag{S24}$$

where $k = 0, 1, \ldots$, such that $S(t_1) = 1$;

(b) When $\rho(0) = |0\rangle\langle 0|$, no t makes $S(t) = 1$.

(2) $|u| > |v|$. (a) When $\rho(0) = |0\rangle\langle 0|$, the solution of t when $S(t) = 1$ are

$$t_2 = kT + \frac{2\hbar}{\Delta}\text{arccot}\left(\pm\frac{2\sqrt{u^2 - r^2\sin^2\theta}}{\Delta}\right), \tag{S25}$$

where $k = 0, 1, \ldots$;

(b) when $\rho(0) = |0\rangle\langle 0|$, $S(t) = 1$ has no solution.

Claim 3. In anti-PPH(real) phase, when $\rho(0) = |+\rangle\langle +|$, if and only if $v = u$, such that the entropy $S(t)$ has an symmetry axis at $t = T/2$ (where T is the period of $S(t)$), i.e. $S(T - t) = S(t)$.
Proof. Solving $m(t)$ when $S(m(T - t)) = S(m(t))$ from Eq. 6 in the manuscript, we have

$$m(T - t) = m(t) \text{ or } m(T - t) = \frac{1}{m(t)} \tag{S26}$$

such that $u = v$, and vice versa. In fact, $S(t) \equiv 1$ in this case when $u = v$.

When $u \neq v$, $S(T - t) \neq S(t)$.

Claim 4. In anti-PPH(real), when $\rho(0) = |+\rangle\langle +|$, the entropy $S(t)$ will reach 1 twice in each period.
Proof. Solving t when $S(t) = 1$, we have

$$t = kT + \frac{2\hbar}{\Delta}\text{arccot}\frac{-u - v}{\Delta} \text{ or } t = kT, \text{ where } k = 0, 1, \ldots \tag{S27}$$

Claim 5. In anti-PPH(imaginary) phase, when $\rho(0) = |0\rangle\langle 0|$, $|1\rangle\langle 1|$, $|+\rangle\langle +|$ or $|-\rangle\langle -|$, the entropy $S(t)$ will approach their asymptotes, respectively.
Proof. When $\rho(0) = |0\rangle\langle 0|$, $|1\rangle\langle 1|$, $|+\rangle\langle +|$ or $|-\rangle\langle -|$ $\lim_{t\to\infty} m(t)$ are

$$\begin{aligned}
m_0 &= m_1 = \frac{v}{u}, \\
m_+ &= \frac{(|\Delta| + 2v)^2 + 4r^2\sin\theta}{(|\Delta| + 2u)^2 + 4r^2\sin\theta}, \\
m_- &= \frac{(|\Delta| - 2v)^2 + 4r^2\sin\theta}{(|\Delta| - 2u)^2 + 4r^2\sin\theta}.
\end{aligned} \tag{S28}$$

Substituting $m(t \to \infty)$'s into Eq. (6), the relevant $S(t \to \infty)$'s are the asymptotes.

Claim 6. In anti-PPH(imaginary) phase, when $\rho(0) = |+\rangle\langle +|$, the entropy will approach to an asymptote $\lim_{t\to\infty} S(t) = S(m_+)$ from above.
Proof. (1) $u + v > 0$, $S(t)$ monotonically deceases to the asymptote $S(m_+)$ from above . (2) $u + v < 0$, there is a moment

$$t_1 = \frac{\hbar}{|\Delta|}\ln\frac{u + v + |\Delta|}{u + v - |\Delta|}, \tag{S29}$$

$S(t)$ will monotonically decease to the asymptote $S(m_+)$ from above after t_1.

Claim 7. In anti-PPH(imaginary) phase, when $\rho(0) = |0\rangle\langle 0|$ or $|1\rangle\langle 1|$, the entropy $S(t)$ will either monotonically increases, or firstly increases to $S = 1$ and then decreasingly approaches to an asymptote.
Proof:

(1) $|u| < |v|$: (a) When $\rho(0) = |0\rangle\langle 0|$, it is easy to check that $S(t)$ will monotonically increase to the asymptote $S(m_0)$ from below; (b) When $\rho(0) = |1\rangle\langle 1|$, there is a moment t_1 such that $S(t)$ increases to $S(t_1) = 1$ from the beginning and then decreases to the asymptote $S(m_1)$ after $t > t_1$, where

$$t_1 = \frac{\hbar}{|\Delta|} \ln \frac{2\sqrt{v^2 - r^2 \sin^2\theta} + |\Delta|}{2\sqrt{v^2 - r^2 \sin^2\theta} - |\Delta|} \tag{S30}$$

(2) $|u| > |v|$: (a) When $\rho(0) = |1\rangle\langle 1|$, $S(t)$ monotonically increases to the asymptote $S(m_0)$ from below; (b) When $\rho(0) = |0\rangle\langle 1|$, $S(t)$ increases to $S(t_1) = 1$ from the beginning to the moment t_1, and then decreases to the asymptote $S(m_0)$ from above, where the

$$t_1 = \frac{\hbar}{|\Delta|} \ln \frac{2\sqrt{u^2 - r^2 \sin^2\theta} + |\Delta|}{2\sqrt{u^2 - r^2 \sin^2\theta} - |\Delta|}. \tag{S31}$$

S3 The entropy dynamics of some non-Hermitian systems to have symmetry under certain initial conditions.

S3.1 PT(real) phase:

For $\rho_\pm(0) = |\psi_\pm(0)\rangle\langle\psi_\pm(0)| = |\pm\rangle\langle\pm| = \frac{1}{2}\begin{pmatrix} 1 & \pm 1 \\ \pm 1 & 1 \end{pmatrix}$, the entropy dynamics is periodic and has a symmetric axis at $t = \frac{T}{2}$. After operated by Eq. S1, the system is evolved to the relevant normalized states and normalized density matrices

$$\begin{aligned}
|\psi_+(t)\rangle &= \frac{1}{\sqrt{k_3}}((a_0 + a_3 + a_1 - ia_2)|0\rangle + (a_0 - a_1 - a_3 - ia_2)|1\rangle)\,, \\
|\psi_-(t)\rangle &= \frac{1}{\sqrt{k_4}}((a_0 - a_1 + a3 + ia_2)0\rangle + (-a_0 - a_1 + a_3 - ia_2)|1\rangle)\,, \\
\rho_+(t) &= \frac{1}{k_3}\begin{pmatrix} (a_0 + a_1 + a_3)^2 + a_2^2 & a_0^2 - (a_1 + a_3 - ia_2)^2 \\ a_0^2 - (a_1 + a_3 + ia_2)^2 & (a_0 - a_1 - a_3)^2 + a_2^2 \end{pmatrix}, \\
\rho_-(t) &= \frac{1}{k_4}\begin{pmatrix} (a_0 - a_1 + a_3)^2 + a_2^2 & -a_0^2 + (-a_1 + a_3 + ia_2)^2 \\ -a_0^2 + (-a_1 + a_3 - ia_2)^2 & (-a_0 - a_1 + a_3)^2 + a_2^2 \end{pmatrix}.
\end{aligned} \tag{S32}$$

where $k_3 = 2a_2^2 + (a_0 + a_1 + a_3)^2 + (a_0 - a_1 - a_3)^2$ and $k_4 = 2a_2^2 + (a_0 - a_1 + a_3)^2 + (-a_0 - a_1 + a_3)^2$. Substituting t by $T - t$, we get $a_0(T - t) = -a_0(t)$ and $a_j(T - t) = a_j(t) (j = 1, 2, 3)$. The time-evolution operator, normalized states and normalized density matrices at moment $T - t$ are

$$\begin{aligned}
e^{-\frac{iH_{PT}(T-t)}{\hbar}} &= e^{-\frac{T-t}{\hbar} ir\cos\theta}\begin{pmatrix} -a_0 + a_3 & a_1 - ia_2 \\ -a_1 - ia_2 & -a_0 - a_3 \end{pmatrix}, \\
|\psi_+(T - t)\rangle &= \frac{1}{\sqrt{k_3}}((-a_0 + a_3 + a_1 - ia_2)|0\rangle + (-a_0 - a_1 - a_3 - ia_2)|1\rangle), \\
|\psi_-(T - t)\rangle &= \frac{1}{\sqrt{k_4}}((-a_0 - a_1 + a3 + ia_2)0\rangle + (a_0 - a_1 + a_3 - ia_2)|1\rangle)\,, \\
\rho_+(T - t) &= \frac{1}{k_3}\begin{pmatrix} (-a_0 + a_1 + a_3)^2 + a_2^2 & a_0^2 - (a_1 + a_3 - ia_2)^2 \\ a_0^2 - (a_1 + a_3 + ia_2)^2 & (-a_0 - a_1 - a_3)^2 + a_2^2 \end{pmatrix}, \\
\rho_-(T - t) &= \frac{1}{k_4}\begin{pmatrix} (-a_0 - a_1 + a_3)^2 + a_2^2 & -a_0^2 + (-a_1 + a_3 + ia_2)^2 \\ -a_0^2 + (-a_1 + a_3 - ia_2)^2 & (a_0 - a_1 + a_3)^2 + a_2^2 \end{pmatrix}.
\end{aligned} \tag{S33}$$

Comparing Eq. S32 with Eq. S33, we can get $\rho_{11}(t) = \rho_{22}(T-t)$ and $\rho_{22}(t) = \rho_{11}(T-t)$ when the initial states are $|\pm\rangle$. It means that the symmetry of the entropy dynamics comes form the normalization of the evolved density matrix with some initial density matrices.

S3.2 Anti-PT(real) phase:

For the initial density matrices $\rho_0(0) = |\psi_0(0)\rangle\langle\psi_0(0)| = |0\rangle\langle 0|$, $\rho_1(0) = |\psi_1(0)\rangle\langle\psi_1(0)| = |1\rangle\langle 1|$, and $\rho_\pm(0) = |\psi_\pm(0)\rangle\langle\psi_\pm(0)| = |\pm\rangle\langle\pm|$, the entropy dynamics is periodical and has an symmetric axis at $t = \frac{T}{2}$. Operated by Eq. S9, the normalized states and normalized density matrices with anti-PT-symmetric Hamiltonians are

$$
\begin{aligned}
|\psi_0(t)\rangle &= \frac{1}{\sqrt{k_1}}((a_0+ia_3)|0\rangle + (a_2-ia_1)|1\rangle)\,,\\
|\psi_1(t)\rangle &= \frac{1}{\sqrt{k_2}}((a_2+ia_1)|0\rangle + (a_0-ia_3)|1\rangle)\,,\\
|\psi_+(t)\rangle &= \frac{1}{\sqrt{k_3}}((a_0+a_2+ia_1+ia_3)|0\rangle + (a_0+a_2-ia_1-ia_3)|1\rangle)\,,\\
|\psi_-(t)\rangle &= \frac{1}{\sqrt{k_4}}((a_0-a_2-ia_1+ia_3)|0\rangle + (-a0+a_2-ia_1+ia_3)|1\rangle)\,,\\
\rho_0(t) &= \frac{1}{k_1}\begin{pmatrix} a_0^2+a_3^2 & (a_0+ia_3)(a_2+ia_1) \\ (a_0-ia_3)(a_2-ia_1) & a_1^2+a_2^2 \end{pmatrix}\,,\\
\rho_1(t) &= \frac{1}{k_2}\begin{pmatrix} a_1^2+a_2^2 & (a_0+ia_3)(a_2+ia_1) \\ (a_0-ia_3)(a_2-ia_1) & a_0^2+a_3^2 \end{pmatrix}\,,\\
\rho_+(t) &= \frac{1}{k_3}\begin{pmatrix} (a_0+a_2)^2+(a_1+a_3)^2 & (a_0+a_2+i(a_1+a_3))^2 \\ (a_0+a_2-i(a_1+a_3))^2 & (a_0+a_2)^2+(a_1+a_3)^2 \end{pmatrix},\\
\rho_-(t) &= \frac{1}{k_4}\begin{pmatrix} (a_0-a_2)^2+(-a_1+a_3)^2 & -(a_0-a_2+i(-a_1+a_3))^2 \\ -(a_0-a_2-i(-a_1+a_3))^2 & (a_0-a_2)^2+(-a_1+a_3)^2 \end{pmatrix}\,.
\end{aligned}
\tag{S34}
$$

where $k_1 = k_2 = a_0^2+a_3^2+a_1^2+a_2^2$, $k_3 = 2(a_0+a_2)^2+2(a_1+a_3)^2$ and $k_4 = 2(a_0-a_2)^2+2(-a_1+a_3)^2$. $t \to T-t$: $a_0(T-t) = -a_0(t)$ and $a_j(T-t) = a_j(t)(j = 1,2,3)$,

$$
\begin{aligned}
e^{-\frac{iH_{APT}(T-t)}{\hbar}} &= e^{\frac{T-t}{\hbar}r\cos\theta}\begin{pmatrix} -a_0+ia_3 & ia_1+a_2 \\ -ia_1+a_2 & -a_0-ia_3 \end{pmatrix}\,,\\
|\psi\rangle_0(T-t) &= \frac{1}{\sqrt{k_1}}((-a_0+ia_3)|0\rangle + (a_2-ia_1)|1\rangle)\,,\\
|\psi\rangle_1(T-t) &= \frac{1}{\sqrt{k_2}}((a_2+ia_1)|0\rangle + (-a_0-ia_3)|1\rangle)\,,\\
|\psi\rangle_+(T-t) &= \frac{1}{\sqrt{k_5}}((-a_0+a_2+ia_1+ia_3)|0\rangle + (-a_0+a_2-ia_1-ia_3)|1\rangle)\,,\\
|\psi\rangle_-(T-t) &= \frac{1}{\sqrt{k_6}}((-a_0-a_2-ia_1+ia_3)|0\rangle + (a0+a_2-ia_1+ia_3)|1\rangle)\,,\\
\rho_0(T-t) &= \frac{1}{k_1}\begin{pmatrix} a_0^2+a_3^2 & (-a_0+ia_3)(a_2+ia_1) \\ (-a_0-ia_3)(a_2-ia_1) & a_1^2+a_2^2 \end{pmatrix}\,,\\
\rho_1(T-t) &= \frac{1}{k_2}\begin{pmatrix} a_1^2+a_2^2 & (-a_0+ia_3)(a_2+ia_1) \\ (-a_0-ia_3)(a_2-ia_1) & a_0^2+a_3^2 \end{pmatrix}\,,\\
\rho_+(T-t) &= \frac{1}{k_5}\begin{pmatrix} (-a_0+a_2)^2+(a_1+a_3)^2 & (-a_0+a_2+i(a_1+a_3))^2 \\ (-a_0+a_2-i(a_1+a_3))^2 & (-a_0+a_2)^2+(a_1+a_3)^2 \end{pmatrix}\,,
\end{aligned}
\tag{S35}
$$

$$\rho_{\_}(T-t)=\frac{1}{k_6}\begin{pmatrix}(-a_0-a_2)^2+(-a_1+a_3)^2 & -(-a_0-a_2+i(-a_1+a_3))^2\\ -(-a_0-a_2-i(-a_1+a_3))^2 & (-a_0-a_2)^2+(-a_1+a_3)^2\end{pmatrix}.$$

where $k_5=2(-a_0+a_2)^2+2(a_1+a_3)^2$ and $k_6=2(a_0+a_2)^2+2(-a_1+a_3)^2$. (i) When the initial state is $|0\rangle$ or $|0\rangle$, we find that $\rho_{kk}(t)=\rho_{kk}(T-t)$ $(k=1,2)$, meaning that the entropy dynamics has a symmetric axis at $t=T/2$. (ii) When the initial state is $|\pm\rangle$, we find that $\rho_{11}(t)=\rho_{22}(t)=1/2$. Therefore, the entropy dynamics are constent.

S3.3 PPH real:

For the initial density matrix $\rho_{\pm}(0)=|\psi_{\pm}(0)\rangle\langle\psi_{\pm}(0)|=|\pm\rangle\langle\pm|$, the entropy dynamics is periodical and has a symmetric axis at $t=T/2$ in each period. Operated by Eq. S13, the normalized states and normalized density matrixes of a P-pseudo-Hermitian Hamiltonian are

$$\begin{aligned}
|\psi_+(t)\rangle &= \frac{1}{\sqrt{k_3}}((a_0+a_3-ia_1)|0\rangle+(a_0-a_3-ia_2)|1\rangle)\,,\\
|\psi_-(t)\rangle &= \frac{1}{\sqrt{k_4}}((a_0+a_3+ia_1)|0\rangle+(-a_0+a_3-ia_2)|1\rangle)\,,\\
\rho_+(t) &= \frac{1}{k_3}\begin{pmatrix}(a_0+a_3)^2+a_1{}^2 & (a_0+a_3-ia_1)(a_0-a_3+ia_2)\\ (a_0-a_3-ia_2)(a_0+a_3+ia_1) & (a_0-a_3)^2+a_2{}^2\end{pmatrix},\\
\rho_-(t) &= \frac{1}{k_4}\begin{pmatrix}(a_0+a_3)^2+a_1{}^2 & (a_0+a_3+ia1)(a_0-a_3+ia_2)\\ (-a_0+a_3-ia_2)(a_0+a_3-ia_1) & (a_0-a_3)^2+a_2{}^2\end{pmatrix}.
\end{aligned}\tag{S36}$$

where $k_3=k_4=a_1^2+a_2^2+2a_0^2+2a_3^2$. $t\to T-t$: $a_0(T-t)=-a_0(t)$ and $a_j(T-t)=a_j(t)(j=1,2,3)$,

$$\begin{aligned}
e^{-\frac{iH_{PPH}(T-t)}{\hbar}} &= e^{-\frac{T-t}{\hbar}ir\cos\theta}\begin{pmatrix}-a_0+a_3 & -ia_1\\ -ia_2 & -a_0-a_3\end{pmatrix},\\
|\psi_+(T-t)\rangle &= \frac{1}{\sqrt{k_3}}((-a_0+a_3-ia_1)|0\rangle+(-a_0-a_3-ia_2)|1\rangle),\\
|\psi_-(T-t)\rangle &= \frac{1}{\sqrt{k_4}}((-a_0+a_3+ia_1)|0\rangle+(a_0+a_3-ia_2)|1\rangle)\,,\\
\rho_+(T-t) &= \frac{1}{k_3}\begin{pmatrix}(-a_0+a_3)^2+a_1{}^2 & (-a_0+a_3-ia_1)(-a_0-a_3+ia_2)\\ (-a_0-a_3-ia_2)(-a_0+a_3+ia_1) & (a_0+a_3)^2+a_2{}^2\end{pmatrix},\\
\rho_{_}(T-t) &= \frac{1}{k_4}\begin{pmatrix}(-a_0+a_3)^2+a_1{}^2 & (-a_0+a_3+ia1)(-a_0-a_3+ia_2)\\ (a_0+a_3-ia_2)(-a_0+a_3-ia_1) & (-a_0+a_3)^2+a_2{}^2\end{pmatrix}.
\end{aligned}\tag{S37}$$

For the initial state $|\pm\rangle$, $\rho_{11}(t)=\rho_{22}(T-t)$ and $\rho_{22}(t)=\rho_{11}(T-t)$. This means that, though the normalized quantum state or density matrix doesn't have the symmetric axis, the entropy dynamics has the symmetric axis at $t=T/2$. Therefore, the symmetry is induced by the normalization of the density matrix only.

S3.4 Anti-PPH-real:

For the initial density matrices $\rho_0(0)=|\psi_0(0)\rangle\langle\psi_0(0)|=|0\rangle\langle 0|$, $\rho_1(0)=|\psi_1(0)\rangle\langle\psi_1(0)|=|1\rangle\langle 1|$, the entropy dynamics is periodical and has a symmetric axis at $t=T/2$. Operated by Eq. S22, the anti-pseudo-Hermitian system evolves to the normalized states and normalized density matrices

$$\begin{aligned}
|\psi_0(t)\rangle &= \frac{1}{\sqrt{k_1}}((a_0+ia_3)|0\rangle+a_2|1\rangle)\,, \qquad |\psi_1(t)\rangle = \frac{1}{\sqrt{k_2}}(a_1|0\rangle+(a_0-ia_3)|1\rangle)\,,\\
\rho_0(t) &= \frac{1}{k_1}\begin{pmatrix}a_0^2+a_3^2 & a_0a_2+ia_2a_3\\ a_0a_2-ia_2a_3 & a_2^2\end{pmatrix}, \qquad \rho_1(t)=\frac{1}{k_2}\begin{pmatrix}a_1^2 & a_0a_1+ia_1a_3\\ a_0a_1-ia_1a_3 & a_0^2+a_3^2\end{pmatrix}.
\end{aligned}\tag{S38}$$

where $k_1=a_0^2+a_2^2+a_3^2$, $k_2=a_0^2+a_1^2+a_3^2$. In order to study the axial symmetry in each period T, we consider the transformation $t\to T-t$. At the beginning, we can determine the changing term $\alpha(T-t)=\pi-\alpha(t)$. Then it's easy to derive $a_0(T-t)=$

$-a_0(t)$ and $a_j(T-t) = a_j(t) (j = 1,2,3)$. We will get the time-evolution operator, normalizing evolving states and normalizing density matrixes in the moment $T-t$ as

$$
e^{-\frac{iH_{APPH}(T-t)}{\hbar}} = e^{\frac{T-t}{\hbar} r\cos\theta} \begin{pmatrix} -a_0 + ia_3 & a_1 \\ a_2 & -a_0 - ia_3 \end{pmatrix} ,
$$

$$
|\psi_0(T-t)\rangle = \frac{1}{\sqrt{k_1}}((-a_0 + ia_3)|0\rangle + a_2|1\rangle) , \qquad |\psi_1(T-t)\rangle = \frac{1}{\sqrt{k_2}}(a_1|0\rangle + (-a_0 - ia_3)|1\rangle) , \tag{S39}
$$

$$
\rho_0(T-t) = \frac{1}{k_1} \begin{pmatrix} a_0^2 + a_3^2 & -a_0a_2 + ia_2a_3 \\ -a_0a_2 - ia_2a_3 & a_2^2 \end{pmatrix} , \qquad \rho_1(T-t) = \frac{1}{k_2} \begin{pmatrix} a_1^2 & -a_0a_1 + ia_1a_3 \\ -a_0a_1 - ia_1a_3 & a_0^2 + a_3^2 \end{pmatrix} .
$$

For the initial states $|0\rangle$ and $|1\rangle$, the diagonal elements of the normalized density matrices satisfy $\rho_{kk}(t) = \rho_{kk}(T-t)$ $(k = 1,2)$, indicating that the entropy dynamics has a symmetric axis at $t = T/2$ which is induced by the specific initial state only.
